# Edaravone use in acute intracerebral hemorrhage: A systematic review and meta-analysis of randomized controlled trials

**DOI:** 10.3389/fphar.2022.935198

**Published:** 2022-08-12

**Authors:** Mingzhen Qin, Luda Feng, Chinyu Yang, Dawei Wei, Tingting Li, Ping Jiang, Jinzhi Guan, Xinyue Zhang, Xinyi Shi, Ning Liang, Xinxing Lai, Li Zhou, Chi Zhang, Ying Gao

**Affiliations:** ^1^ Institute for Brain Disorders, Beijing University of Chinese Medicine, Beijing, China; ^2^ Department of Neurology, Dongzhimen Hospital, Beijing University of Chinese Medicine, Beijing, China; ^3^ Beijing University of Chinese Medicine, Beijing, China; ^4^ Dongfang Hospital, Beijing University of Chinese Medicine, Beijing, China; ^5^ Department of Cardiology, Dongzhimen Hospital, Beijing University of Chinese Medicine, Beijing, China; ^6^ Department of Rheumatology, Guang’anmen Hospital, China Academy of Chinese Medical Sciences, Beijing, China; ^7^ Department of Acupuncture and Moxibustion, Beijing Hospital of Traditional Chinese Medicine, Capital Medical University, Beijing, China; ^8^ Institute of Basic Research in Clinical Medicine, China Academy of Chinese Medical Sciences, Beijing, China; ^9^ Dongzhimen Hospital, Beijing University of Chinese Medicine, Beijing, China; ^10^ Chinese Medicine Key Research Room of Brain Disorders Syndrome and Treatment of the National Administration of Traditional Chinese Medicine, Beijing, China

**Keywords:** intracerebral hemorrhage, edaravone, mortality, neurological deficits, systematic review, meta-analysis

## Abstract

**Background:** Edaravone alleviates neurological deficits among patients with intracerebral hemorrhage; however, its effects on mortality and long-term functional outcomes remain unknown.

**Objective:** To assess clinical outcomes associated with edaravone initiated within 7 days of symptoms onset in intracerebral hemorrhage.

**Methods:** We systematically searched PubMed, Embase, Cochrane Library, CiNii, China National Knowledge Infrastructure, Chinese VIP information, Wanfang Data, and SinoMed for relevant randomized controlled trials from their inception to 1 May 2021 and conducted a comprehensive systematic review and meta-analysis (PROSPERO registration number: CRD42019147801). All-cause mortality and long-term functional outcomes were taken as the primary outcomes.

**Results:** A total of 38 randomized controlled trials including 3,454 participants with acute intracerebral hemorrhage were included. The selected articles were of poor quality. Meta-analysis revealed that edaravone could not reduce all-cause mortality [relative risk (RR) = 0.51; 95% confidence interval (CI) (0.11–2.32); *p* = 0.38]. No studies reported on long-term functional outcomes in those trials. In addition, edaravone alleviated neurological deficits [mean difference (MD) = −5.44; 95% CI (−6.44 to −4.44); *p*<0.00001], improved the activities of daily living [MD = 8.44; 95% CI (7.65–9.23); *p*<0.00001], reduced the hematoma volume [MD = −4.71; 95% CI (−5.86 to −3.56); *p*<0.00001], and increased treatment response [RR = 1.26; 95% CI (1.22–1.31); *p*<0.00001]. In terms of safety outcome, there was no significant difference between the edaravone group and the control groups [RR = 1.67; 95% CI (0.92 to 3.06); *p* = 0.09].

**Conclusion:** Till date, edaravone does not associate with mortality reduction when initiated within 7 days of intracerebral hemorrhage onset. The effect of edaravone on long-term functional outcomes remains unknown due to lack of data. Although edaravone alleviated neurological deficits, improved activities of daily living, and reduced hematoma volume, we cautiously interpreted the results owing to the overall poor quality and high heterogeneity of the included trials. Presently, the results are insufficient to support edaravone as a routine treatment option for acute intracerebral hemorrhage.

## 1 Introduction

Acute spontaneous, non-traumatic intracerebral hemorrhage (ICH) is a life-threatening disease associated with a mean of 9.46 disability-adjusted life-years, which is defined as the combination of years of life lost and years lived with disability, and bring about enormous health care and economic burden (Steiner et al., 2014; Cordonnier et al., 2018; GBD 2019 Diseases and Injuries Collaborators, 2020; Cao et al., 2020; Haupenthal et al., 2021). Currently, neuroprotection of the peripheral injured brain tissue, which is widely used by clinical researchers and doctors, is a rational but unproven approach to improve clinical outcomes of patients with acute ICH (Hemphill et al., 2015).

The pathological mechanisms of ICH can generally be divided into primary injuries caused by the physical damage of hematoma and secondary injuries triggered by the extravasated blood components and the disruption of mitochondria and intrinsic antioxidant systems (Keep et al., 2012; Shang et al., 2015). Oxidative stress is prominently involved in secondary injuries due to the massive oxidative damage to proteins, nucleic acids, carbohydrates, and lipids (Xi et al., 2006; Aronowski and Zhao, 2011; Shang et al., 2015). The effect of antioxidants to reduce ICH-induced brain injury has also been confirmed in animal models (Peeling et al., 1998; Peeling et al., 2001).

Edaravone (MCI-186, 3-methyl-1-phenyl-2-pyrazolin-5-one) is a free radical scavenger which can not only reduce oxidative DNA damage by reducing apurinic/apyrimidinic sites and 8-OHdG levels in rat models with acute ICH but also attenuate brain edema and ameliorate neurologic deficits by reducing iron- and thrombin-induced injury as well as suppressing NLRP3 in microglia (Nakamura et al., 2008; Shang et al., 2015; Zhang et al., 2016a; Miao et al., 2020). Edaravone is widely used in China and India and has been adopted by the Chinese Guidelines for Diagnosis and Treatment of Acute Intracerebral Hemorrhage 2019 with a weak recommendation (Class Ⅱ, level of evidence C) (Yang et al., 2011; Chinese Society of Neurology, and Chinese Stroke Society, 2019).

Nevertheless, the aforementioned recommendation was drawn on the unrobust results of previous systematic reviews, suggesting that edaravone could exert slightly promising effects on the neurological deficit improvement of ICH (Yang et al., 2011; Yang et al., 2015). The conclusive effects of edaravone treatment on survival or long-term functional outcomes in patients with ICH remain unclear. Recent reviews reveal that several randomized controlled trials (RCTs) have demonstrated that edaravone could not only improve the activities of daily living but also reduce the hematoma volume and the edema zone without increasing mortality and adverse events. Furthermore, several RCTs have reported long-term fatality and functional status, as well as adverse reactions associated with edaravone use (Abe et al., 2007).

Taken together, we conducted this updated systematic review and meta-analysis to evaluate the efficacy and safety of edaravone in the treatment of acute ICH to obtain conclusive evidence and provide clinicians and patients with the latest evidence-based options for edaravone use in ICH.

## 2 Materials and methods

The systematic review and meta-analysis were reported in accordance with the Preferred Reporting Items for Systematic Reviews and Meta-Analyses (PRISMA) reporting guidelines (Page et al., 2021). We also used the Grading of Recommendations Assessment, Development and Evaluation (GRADE) system to evaluate the certainty of the evidence derived from the meta-analysis results (Guyatt et al., 2011a; Guyatt et al., 2011b; Guyatt et al., 2011c; Guyatt et al., 2011d; Guyatt et al., 2011e). This study was conducted according to our previously published protocol (CRD42019147801) (Feng et al., 2020).

### 2.1 Search strategy and study screening

We conducted a comprehensive search on PubMed, Embase, Cochrane Library, CiNii, China National Knowledge Infrastructure (CNKI), Chinese VIP information (VIP), Wanfang Data, and SinoMed for relevant randomized controlled trials from their respective inception dates to 1 May 2021. All searches were conducted by combining free-text and MESH terms, and the following terms were used: “cerebral hemorrhage,” “edaravone,” and “randomized controlled trial” (Supplementary Table S1). The registered clinical trials, ongoing or unpublished trials, dissertations, and gray literature were also searched, regardless of language limitations. A secondary manual search was also performed based on the references of the included studies. After removing duplicate studies, two reviewers (Feng L. and Jiang P.) screened the articles together through abstracts and full texts successively, to assess eligibility.

### 2.2 Inclusion and exclusion criteria

We included RCTs that initiated edaravone within 7 days of symptom onset and determined its efficacy and safety compared with no treatment or placebo. In addition, trials wherein conventional treatments, surgeries, and other co-interventions with edaravone were administered equally to all groups were also included. Non-RCTs, animal studies, reviews, commentaries, and meta-analyses were excluded from this study. In addition, studies on patients with traumatic hemorrhage, primary intraventricular hemorrhage, or subarachnoid hemorrhage were also excluded.

### 2.3 Data extraction

Information from the eligible RCTs was independently extracted by reviewers, in pairs (Qin M., Guan J., Zhang X., and Shi X.), using a preformulated data collection form which included authors, publication year, number of participating sites, sample size, patient characteristics, intervention details, and outcomes. The primary outcomes were all-cause mortality and unfavorable functional outcomes, defined as modified Rankin Scale (mRS) grades 3–6, Glasgow Outcome Scale (GOS) grades 1–3, or Barthel Index (BI) less than or equal to 60 at the end of the follow-up. The secondary outcomes included neurological impairments, which were assessed using clinical scales including the National Institutes of Health Stroke Scale (NIHSS), Canadian Neurological Scale (CNS), European Stroke Scale (ESS), Scandinavian Stroke Scale (SSS), and Modified Edinburgh-Scandinavian Stroke Scale (MESSS) as well as other related scales, and the activities of daily living measured with BI, hematoma volume, and the total efficiency rate calculated by the ratio of effective number to total number after treatment. Edaravone-induced adverse events included liver impairment, kidney impairment, skin irritation, and nausea.

### 2.4 Risk of bias assessment and grading of evidence

Two reviewers (Yang C. and Wei D.) independently assessed the risk of bias of all included studies using the Cochrane Risk of Bias Tool. The GRADE system was used for each meta-analysis to ascertain the overall strength of evidence across the trials, and two reviewers (Qin M. and Li T.) downgraded evidence based on the risk of bias, inconsistency, indirectness, imprecision, and publication bias. Disagreements were resolved by discussion or by involving another review author (Gao Y.) to arbitrate.

### 2.5 Data synthesis and analysis

Statistical analyses were performed using STATA 16.0 and Revman 5.4 software. We calculated the relative risk (RR) from the number of events and participants and mean difference (MD) from the number of participants as well as the mean and standard deviation (SD) after treatment in each group. The effect estimates of continuous data (neurological deficits, activities of daily living, and hematoma volume) were measured by MD with a 95% confidence interval (CI), whereas RR with 95% CI was adopted for dichotomous data (mortality, total efficiency rate, and adverse events). Heterogeneity between studies was calculated using *I* ^*2*^ statistics. We calculated the pooled effect size using the fixed effect model when *I* ^*2*^ was less than 25% and the random effect or qualitative analysis model when *I* ^*2*^ was 25% or greater. A two-tailed *p* < 0.05 was considered statistically significant. A sensitivity analysis was performed by omitting each study one at a time to calculate the pooled effect size. A Subgroup analysis was performed according to the severity, co-intervention, duration of treatment, and dose range of edaravone per day. The potential publication bias was assessed by visual inspection of funnel plot symmetry and validated by Egger’s test.

## 3 Results

### 3.1 Search results

**FIGURE 1:**
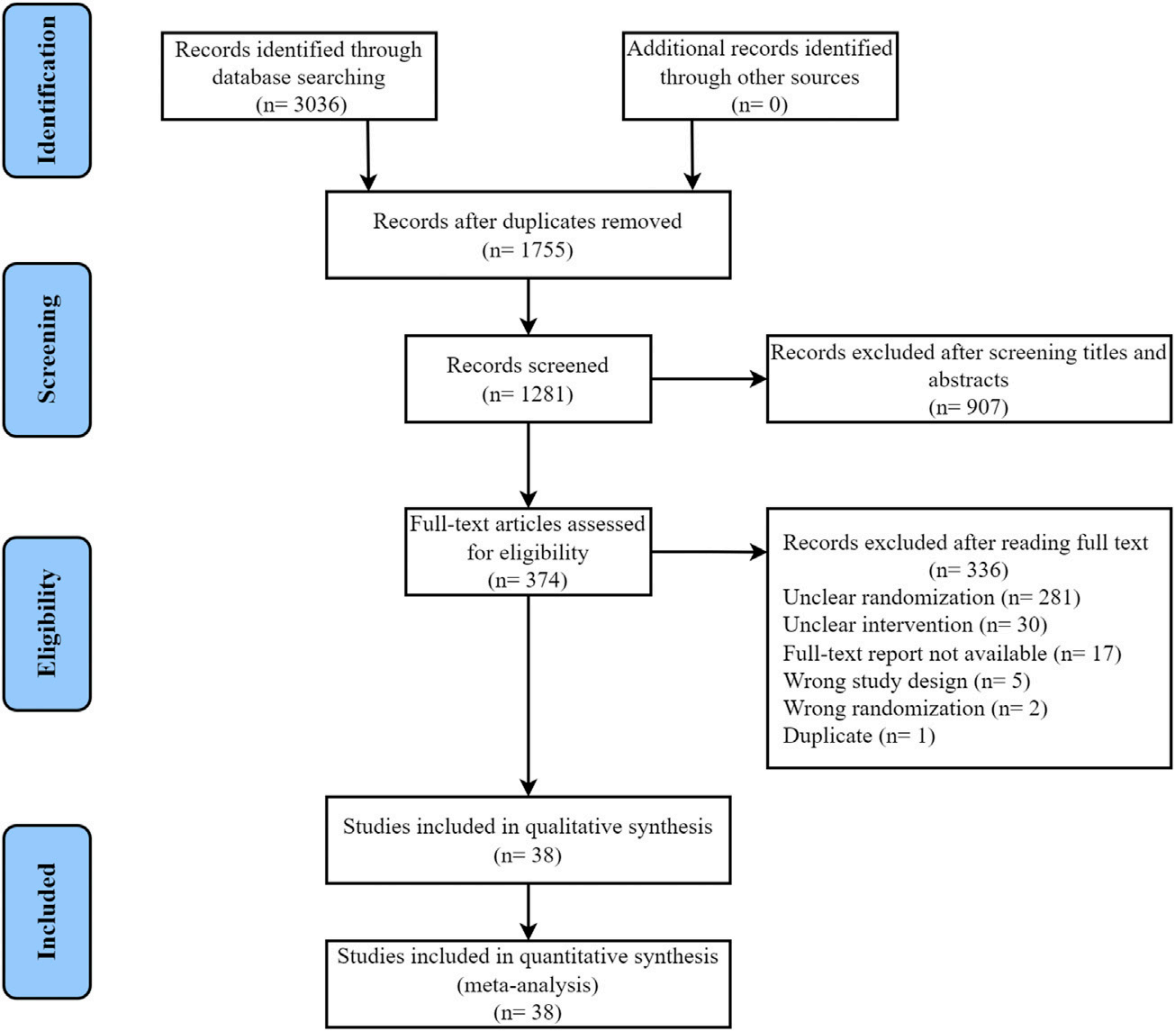
Flow diagram of study selection.

A total of 3,036 articles were obtained by our systematic search, 1,755 of which were excluded owing to duplications and 907 were excluded for inappropriate research type and content after reviewing the title or abstract. A total of 374 articles were subjected to full-text review. Of these, 336 studies were excluded for inappropriate study design, including unclear randomization (281 articles), unclear intervention (30 articles), unavailable full-text report (17 articles), wrong study design (5 articles), wrong randomization (2 articles), and duplicate (1 article). A total of 38 relevant studies were included in the final dataset, and the literature search and article selection are depicted in Figure 1 (Geng et al., 2004; Li and Shan, 2005; Wang et al., 2007; He et al., 2009a; He et al., 2009b; Hao, 2009; Liu, 2009; Zhao et al., 2009; Wang et al., 2010a; Wang et al., 2010b; Ma and Qiao, 2010; Sang and Gong, 2010; Wu, 2011; Zhao et al., 2011; Zhou, 2011; Liang and Zhou, 2013; Zhan et al., 2013; He, 2014; Qu and Wei, 2015; Ran, 2015; Zhang et al., 2016b; Duan and Cao, 2016; Jiang, 2016; Li et al., 2016; Sun et al., 2016; Tian and Pan, 2016; Cheng, 2017; Guo et al., 2017; Zhang et al., 2017; Li et al., 2018; Wan, 2018; Wang et al., 2018; Chen et al., 2019; Xu et al., 2019; Chi, 2020; Li et al., 2020; Wang, 2020; Ye et al., 2020).

### 3.2 Characteristics of the included studies

Overall, 38 studies involving 3,454 participants were included, all of which were single-center studies conducted in mainland China. The characteristics of these 38 studies are listed in Table 1. The sample size ranged from 58 to 190, and the mean age ranged from 45.8 to 71.03 years. The patients were admitted to the hospital within 72 h of ICH onset, and the average bleeding volume was 9–66 ml, according to the image findings. The dose range of edaravone was 30 mg or 60 mg per day, and the duration of treatment was 14–30 days. The co-interventions included conventional treatment, surgery, or neuroprotective agents. Only one study conducted placebo control, whereas the others added on co-interventions.

**TABLE 1:** Characteristics of included studies in the final meta-analysis.

^Source^	^Study site^	^Sample size^	^Patients no. (Tre/Con)^	^Time window (h)^	^Dose range (mg/day)^	^Duration of treatment (days (d))^	^Combination treatment^	^Primary outcome^	^Safety outcome^
^Chen et al. (2019)^	^China^	^76^	^38/38^	^<24^	^30^	^30^	^CT, nimodipine^	^NIHSS^	^NR^
^Cheng (2017)^	^China^	^78^	^39/39^	^<24^	^60^	^14^	^CT^	^TER^	^Nausea and vomiting^
^Chi, (2020)^	^China^	^70^	^35/35^	^<21^	^60^	^14^	^CT and nimodipine^	^NIHSS^	^NR^
^Duan and Cao, (2016)^	^China^	^60^	^30/30^	^<24^	^60^	^14^	^CT and MNF^	^NIHSS^	^NR^
^Geng et al. (2004)^	^China^	^58^	^29/29^	^<72^	^60^	^28^	^CT^	^CSS^	^Palpitating^
^Guo et al. (2017)^	^China^	^146^	^73/73^	^<48^	^60^	^14^	^CT and surgery^	^NIHSS^	^NR^
^Hao (2009)^	^China^	^72^	^36/36^	^<48^	^60^	^14^	^CT^	^TER^	^NR^
^He et al. (2009a)^	^China^	^62^	^31/31^	^<72^	^60^	^14^	^CT^	^Mortality^	^Palpitating^
^He et al. (2009b)^	^China^	^73^	^38/35^	^<72^	^60^	^14^	^CT^	^Mortality and NIHSS^	^Kidney impairment, skin irritation, and pruritus^
^He (2014)^	^China^	^145^	^72/73^	^<24^	^60^	^14^	^CT^	^TER^	^NR^
^Jiang (2016)^	^China^	^78^	^39/39^	^<6^	^30^	^30^	^CT^	^NIHSS^	^NR^
^Li and Shan, (2005)^	^China^	^116^	^58/58^	^<72^	^60^	^19^	^CT^	^TER^	^NR^
^Li et al. (2016)^	^China^	^80^	^40/40^	^<24^	^NR^	^14^	^CT^	^NIHSS^	^NR^
^Li et al. (2018)^	^China^	^80^	^40/40^	^<48^	^60^	^14^	^CT^	^NIHSS^	^NR^
^Li et al. (2020)^	^China^	^190^	^95/95^	^<24^	^60^	^14^	^CT and MNF^	^NIHSS^	^NR^
^Liang and Zhou (2013)^	^China^	^120^	^60/60^	^<72^	^60^	^14^	^CT^	^NIHSS^	^NR^
^Liu (2009)^	^China^	^72^	^36/36^	^<48^	^60^	^14^	^CT^	^NIHSS and GOS^	^NR^
^Ma and Qiao, (2010)^	^China^	^64^	^32/32^	^<72^	^60^	^14^	^CT^	^TER^	^NR^
^Qu and Wei, (2015)^	^China^	^60^	^30/30^	^<36^	^60^	^14^	^CT^	^CSS^	^Liver/kidney impairment and nausea^
^Ran (2015)^	^China^	^88^	^44/44^	^<24^	^60^	^14^	^CT^	^NIHSS^	^NR^
^Sang and Gong, (2010)^	^China^	^64^	^32/32^	^<72^	^60^	^14^	^CT^	^BI^	^NR^
^Sun et al. (2016)^	^China^	^84^	^42/42^	^<72^	^60^	^14^	^CT^	^HV^	^NR^
^Tian and Pan, (2016)^	^China^	^104^	^52/52^	^<6^	^60^	^14^	^CT^	^NIHSS^	^NR^
^Wan, (2018)^	^China^	^120^	^60/60^	^<24^	^60^	^14^	^CT^	^NIHSS^	^NR^
^Wang et al. (2007)^	^China^	^71^	^36/35^	^<48^	^60^	^14^	^CT^	^NIHSS^	^Liver/kidney impairment and skin irritation^
^Wang et al. (2010a)^	^China^	^78^	^39/39^	^<48^	^60^	^14^	^CT^	^NIHSS^	^NR^
^Wang et al. (2010b)^	^China^	^70^	^35/35^	^<24^	^60^	^14^	^CT and surgery^	^BI^	^NR^
^Wang et al. (2018)^	^China^	^86^	^43/43^	^<24^	^60^	^14^	^CT and nimodipine^	^NIHSS^	^NR^
^Wang, (2020)^	^China^	^73^	^37/36^	^<24^	^60^	^14^	^CT^	^NIHSS^	^NR^
^Wu (2011)^	^China^	^64^	^32/32^	^<48^	^60^	^14^	^CT and surgery^	^TER^	^Liver impairment^
^Xu et al. (2019)^	^China^	^120^	^60/60^	^<72^	^60^	^30^	^CT and nimodipine^	^NIHSS^	^NR^
^Ye et al. (2020)^	^China^	^98^	^49/49^	^<24^	^60^	^14^	^CT and surgery^	^TER^	^Liver impairment and skin irritation^
^Zhan et al. (2013)^	^China^	^160^	^80/80^	^<24^	^60^	^14^	^CT and placebo^	^NIHSS^	^NR^
^Zhang et al. (2016a)^	^China^	^98^	^50/48^	^<72^	^60^	^14^	^CT and surgery^	^NIHSS^	^NR^
^Zhang et al. (2017)^	^China^	^72^	^36/36^	^<48^	^60^	^28^	^CT and surgery^	^NIHSS^	^NR^
^Zhao et al. (2009)^	^China^	^72^	^36/36^	^<48^	^60^	^14^	^CT^	^NIHSS^	^NR^
^Zhao et al. (2011)^	^China^	^138^	^73/65^	^<24^	^60^	^14^	^CT and surgery^	^NIHSS^	^NR^
^Zhou (2011)^	^China^	^94^	^47/47^	^<24^	^60^	^14^	^CT^	^TER^	^NR^

Abbreviations: Tre, treatment group; Con, control group; CT, conventional treatment; NR, not reported; MNF, mouse nerve factor injection; TER, total efficiency rate; HV, hematoma volume.

**FIGURE 2:**
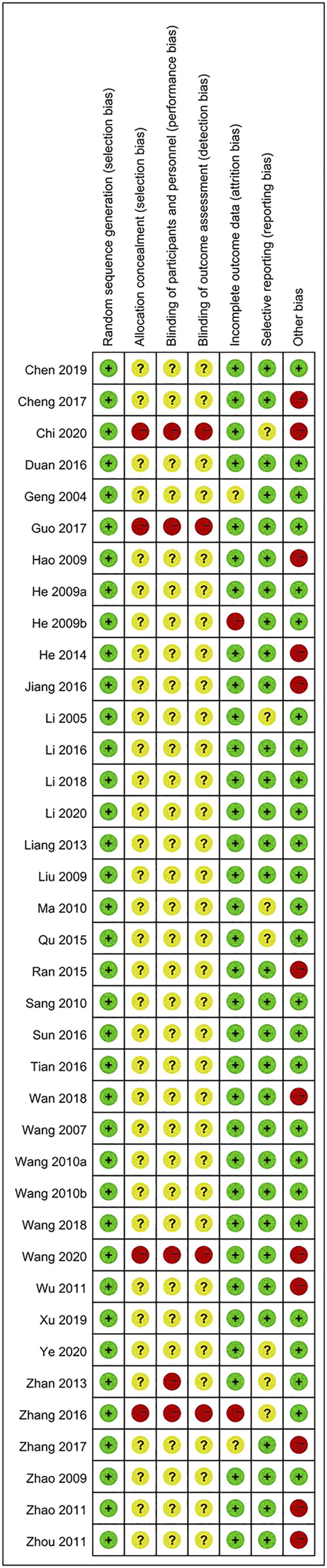
Risk of bias summary for each study.

Evaluation of bias risk for all articles is presented in Figure 2. The risk of bias in the RCTs was examined based on seven items. Most of the included articles did not meet these standards, indicating that the selected articles were of poor quality.

### 3.3 Primary outcomes

#### 3.3.1 All-cause mortality

**FIGURE 3:**
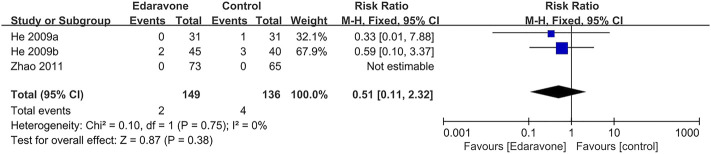
Forest plot for the effect of edaravone on all-cause mortality.

Three articles (He et al., 2009a; He et al., 2009b; Zhao et al., 2011) provided no evidence proving that edaravone could reduce the all-cause mortality of acute ICH (RR = 0.51; 95% CI [0.11 to 2.32]; *p* = 0.38; *I* ^*2*^ = 0%) (Figure 3).

#### 3.3.2 Long-term functional outcomes

One of the studies reported the long-term functional outcome with BI at 60 days; however, its random sequence generation and onset time were unknown. Therefore, we excluded this study (Ze, 2015).

### 3.4 Secondary outcomes

#### 3.4.1 Improvement of neurological impairment

**FIGURE 4:**
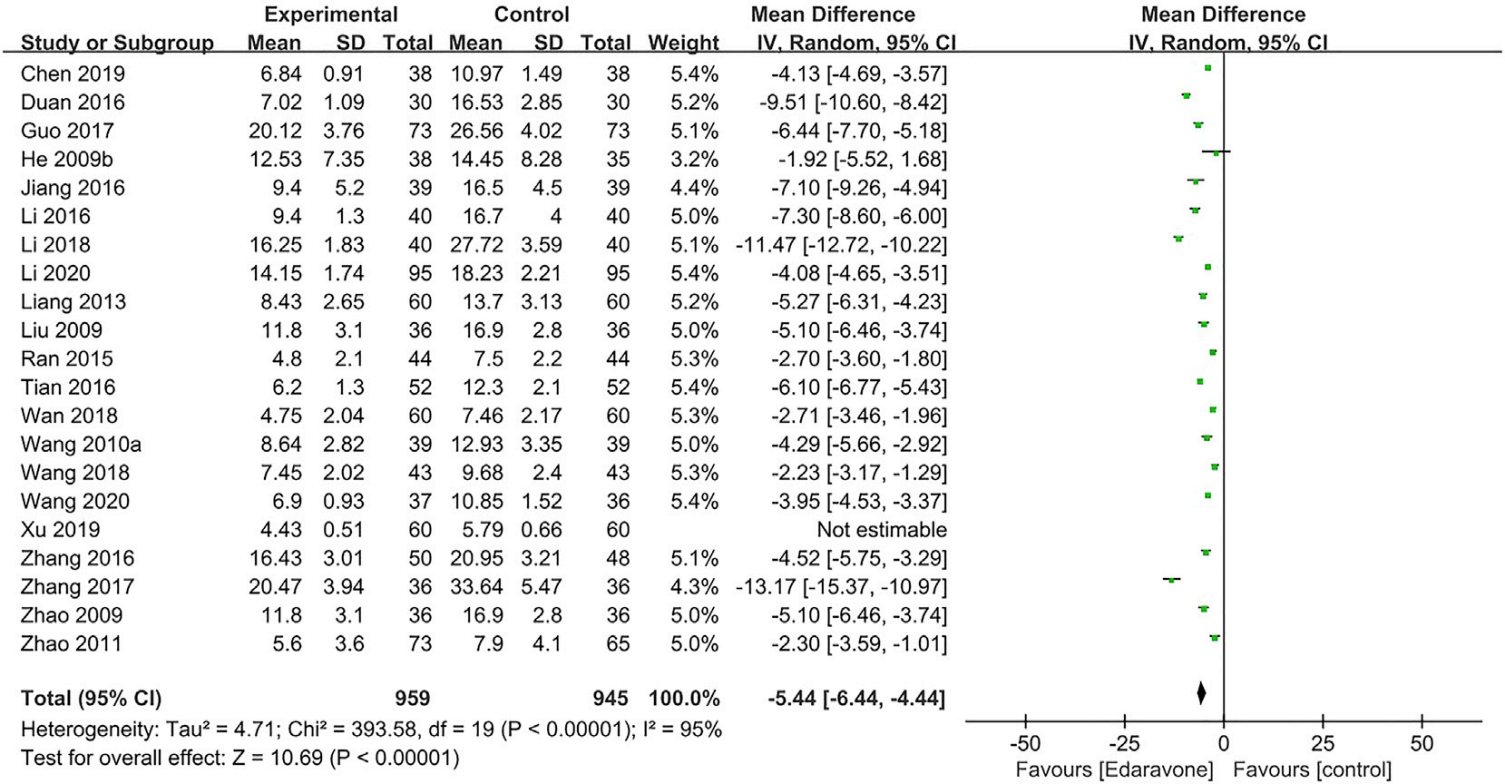
Forest plot for the effect of edaravone on the NIHSS score.

A total of 21 articles (He et al., 2009b; Liu, 2009; Zhao et al., 2009; Wang et al., 2010a; Zhao et al., 2011; Liang and Zhou, 2013; Ran, 2015; Zhang et al., 2016a; Duan and Cao, 2016; Jiang, 2016; Li et al., 2016; Tian and Pan, 2016; Guo et al., 2017; Zhang et al., 2017; Li et al., 2018; Wan, 2018; Wang et al., 2018; Chen et al., 2019; Xu et al., 2019; Li et al., 2020; Wang, 2020) used the NIHSS, and the pooled data illustrated that edaravone could reduce the NIHSS score [MD = −5.44; 95% CI (−6.44 to −4.44); *p* < 0.00001] (Figure 4). Considering the high heterogeneity in the meta-analysis of the NIHSS score (*I* ^*2*^ = 95%, *p* < 0.00001), we further performed a sensitivity analysis, and one study was excluded (Xu et al., 2019). Subgroup analyses were also conducted, respectively, by severity [moderate-to-severe was defined as the NIHSS score 5–25 before treatment, MD = −4.32; 95% CI (−5.12 to −3.52); *p* < 0.00001; *I* ^*2*^ = 89%; severe was defined as the NIHSS score above 25 before treatment, MD = −8.13; 95% CI (−11.00 to −5.26); *p* < 0.00001; *I* ^*2*^ = 98%] (Supplementary Figure S1), by co-intervention [conventional treatment, MD = −4.95; 95% CI (−6.38 to −3.53); *p* < 0.00001; *I* ^*2*^ = 95%; conventional treatment plus surgery, MD = −4.42; 95% CI (−6.74 to −2.11); *p =* 0.0002; *I* ^*2*^ = 90%; other co-interventions, MD = −6.64; 95% CI (−8.63 to −4.64); *p* < 0.00001; *I* ^*2*^ = 97%] (Supplementary Figure S2), by duration of treatment (14 days, MD = −5.05; 95% CI (−6.13 to −3.97); *p* < 0.00001; *I* ^*2*^ = 95%; 28 days, MD = −13.17; 95% CI (−15.37 to −10.97); *p* < 0.00001; *I* ^*2*^ = not applicable; 30 days, MD = −5.42; 95% CI (−8.31 to −2.54); *p =* 0.0002; *I* ^*2*^ = 85%] (Supplementary Figure S3), and by dose range of edaravone per day [30 mg, MD = −5.42; 95% CI (−8.31 to −2.54); *p* = 0.0002; *I* ^*2*^ = 85%; 60 mg, MD = −5.33; 95% CI (−6.49 to −4.18); *p* < 0.00001; *I* ^*2*^ = 96%] (Supplementary Figure S4).

#### 3.4.2 Improvement of activities of daily living

**FIGURE 5:**
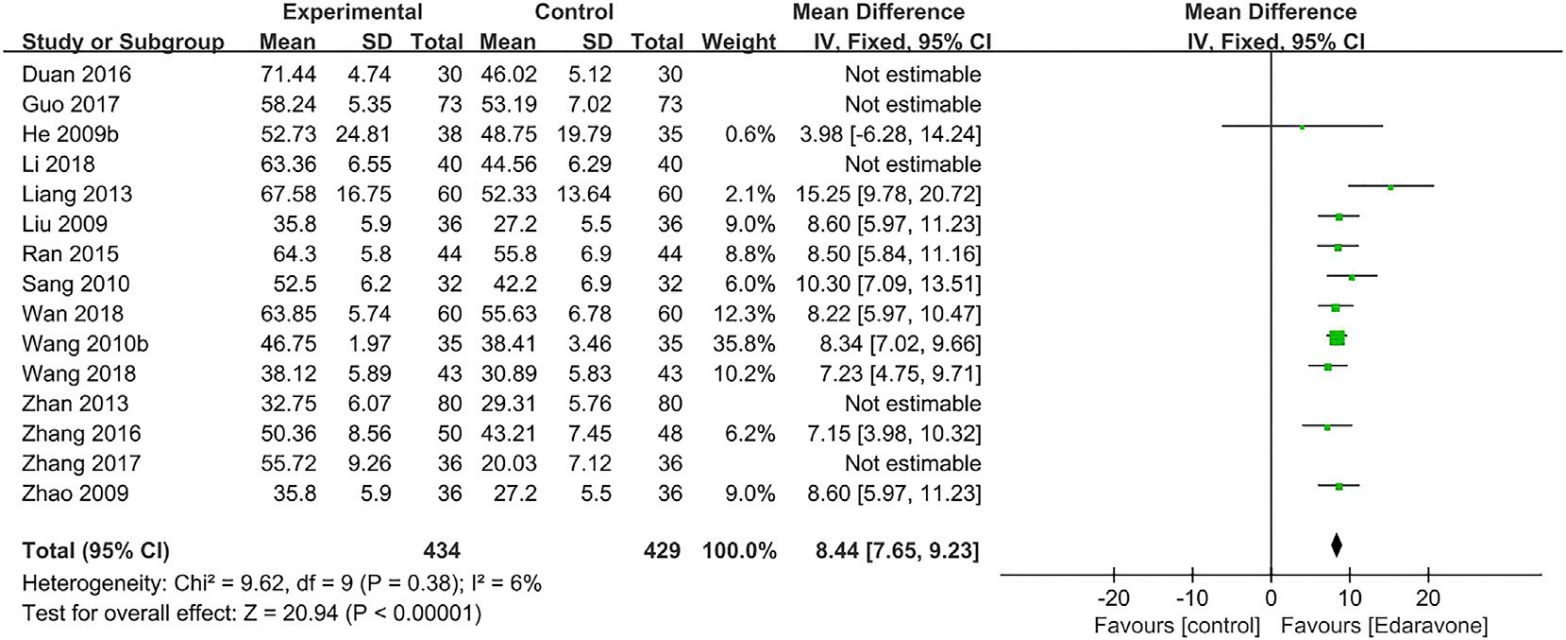
Forest plot for the effect of edaravone on BI.

As for the activities of daily living, 15 articles (He et al., 2009b; Liu, 2009; Zhao et al., 2009; Wang Z. et al., 2010; Sang and Gong, 2010; Liang and Zhou, 2013; Zhan, et al., 2013; Ran, 2015; Zhang et al., 2016b; Duan and Cao, 2016; Guo et al., 2017; Zhang et al., 2017; Li et al., 2018; Wan, 2018; Wang et al., 2018) reported the grading according to BI. Considering the high heterogeneity in BI (*I* ^*2*^ = 95%, *p* < 0.00001), we further conducted a sensitivity analysis, and five studies were removed (Zhan, et al., 2013; Duan and Cao, 2016; Guo et al., 2017; Zhang et al., 2017; Li et al., 2018). The outcome indicated that edaravone could improve the BI [MD = 8.44; 95% CI (7.65–9.23); *p* < 0.00001; *I* ^*2*^ = 6%] (Figure 5).

#### 3.4.3 Reduction of hematoma volume

**FIGURE 6:**
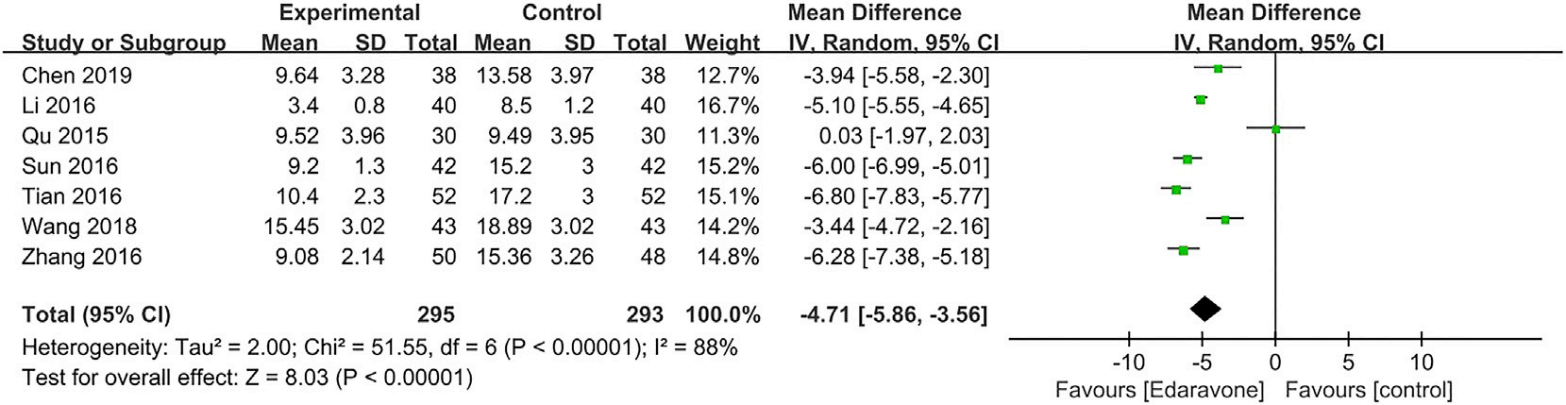
Forest plot for the effect of edaravone on hematoma volume.

A total of seven articles (Qu and Wei, 2015; Zhang et al., 2016a; Li et al., 2016; Sun et al., 2016; Tian and Pan, 2016; Wang et al., 2018; Chen et al., 2019) reported the hematoma volume, and the pooled data showed that edaravone could reduce the hematoma volume [MD = −4.71; 95% CI (−5.86 to −3.56); *p* < 0.00001] (Figure 6). Since high heterogeneity existed (*I* ^*2*^ = 88%, *p* < 0.00001), we conducted a sensitivity analysis, and no study was removed. Subgroup analyses were performed as well, and we divided it into four subgroups, respectively, by severity (moderate-to-severe, MD = −4.91; 95% CI (−6.17 to −3.65); *p* < 0.00001; *I* ^*2*^ = 84%; severe, MD = −6.28; 95% CI [−7.38 to −5.18]; *p* < 0.00001; *I* ^*2*^ = not applicable] (Supplementary Figure S5), by co-intervention [by conventional treatment, MD = −4.40; 95% CI (−7.46 to −1.35); *p =* 0.005; *I* ^*2*^ = 94%; by convention treatment plus surgery, MD = −6.28; 95% CI (−7.38 to −5.18); *p* < 0.00001; *I* ^*2*^ = not applicable; by other co-interventions, MD = −4.30; 95% CI (−5.49 to −3.12); *p* < 0.00001; *I* ^*2*^ = 72%] (Supplementary Figure S6), by duration of treatment [14 days, MD = −4.81, 95% CI (−6.07 to −3.55); *p* < 0.00001; *I* ^*2*^ = 90%; 30 days, MD = −3.94; 95% CI (−5.58 to −2.30); *p* < 0.00001; *I* ^*2*^ = not applicable] (Supplementary Figure S7), and by dose range of edaravone per day [30 mg, MD = −3.94; 95% CI (−5.58 to −2.30); *p* < 0.00001; *I* ^*2*^ = not applicable; 60 mg, MD = −4.64; 95% CI (−6.51 to −2.78); *p* < 0.00001; *I* ^*2*^ = 92%] (Supplementary Figure S8).

#### 3.4.4 Total efficiency rate

**FIGURE 7:**
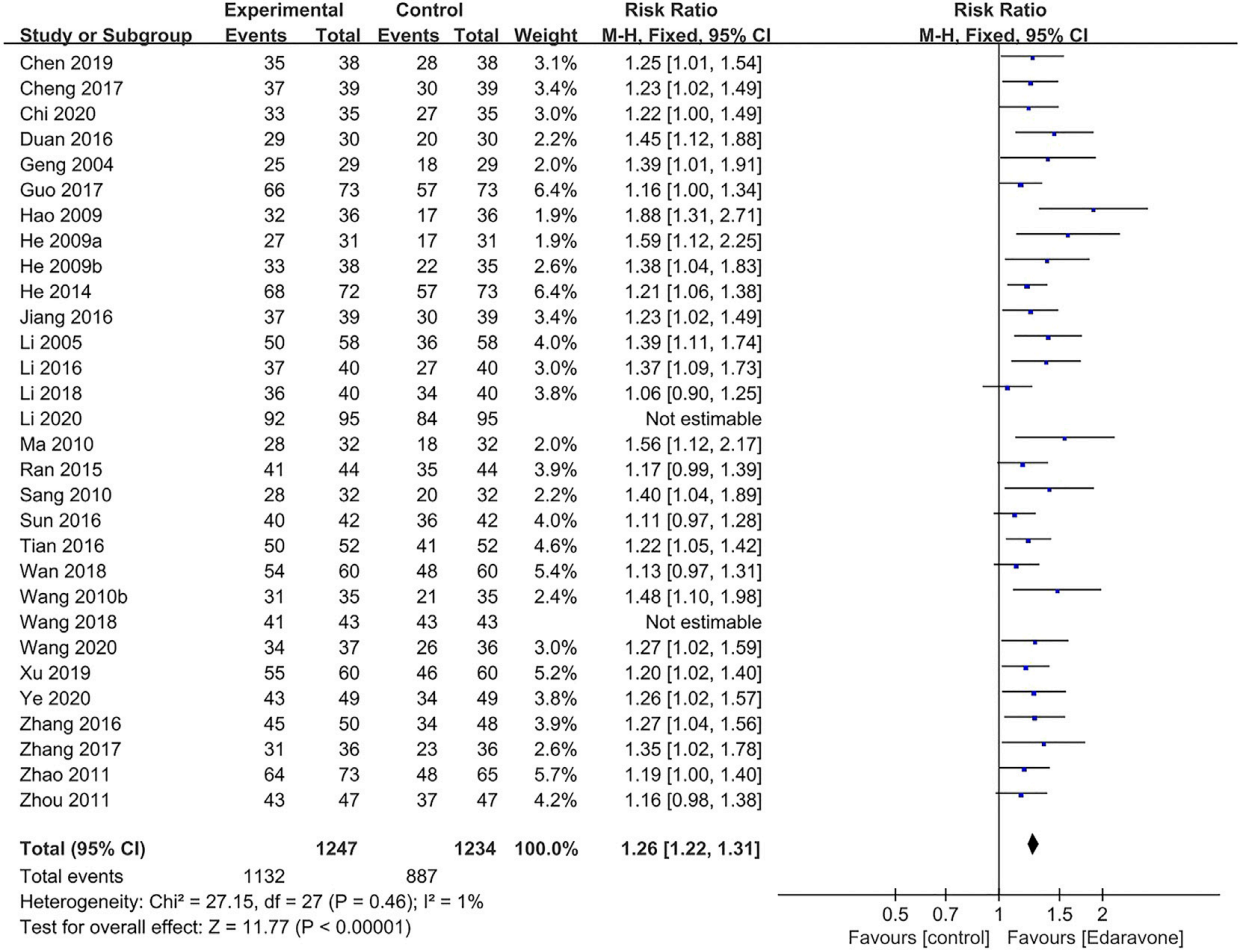
Forest plot for the effect of edaravone on the total efficiency rate.

A total of 30 articles (Geng et al., 2004; Li and Shan, 2005; He et al., 2009a; He et al., 2009b; Hao, 2009; Wang et al., 2010b; Ma and Qiao, 2010; Sang and Gong, 2010; Zhao et al., 2011; Zhou, 2011; He, 2014; Ran, 2015; Zhang et al., 2016b; Duan and Cao, 2016; Jiang, 2016; Li et al., 2016; Sun et al., 2016; Tian and Pan, 2016; Cheng, 2017; Guo et al., 2017; Zhang et al., 2017; Li et al., 2018; Wan, 2018; Wang et al., 2018; Chen et al., 2019; Xu et al., 2019; Chi, 2020; Li et al., 2020; Wang, 2020; Ye et al., 2020) reported the total efficiency rate at the end of follow-up, while two of them were excluded in the sensitivity analysis as meta-analyses could not be performed (Wang et al., 2018; Li et al., 2020). The outcomes showed that edaravone was more likely to improve neurological impairment [RR = 1.26; 95% CI (1.22–1.31); *p* < 0.00001; *I* ^*2*^ = 1%] (Figure 7).

#### 3.4.5 Adverse events

**FIGURE 8:**
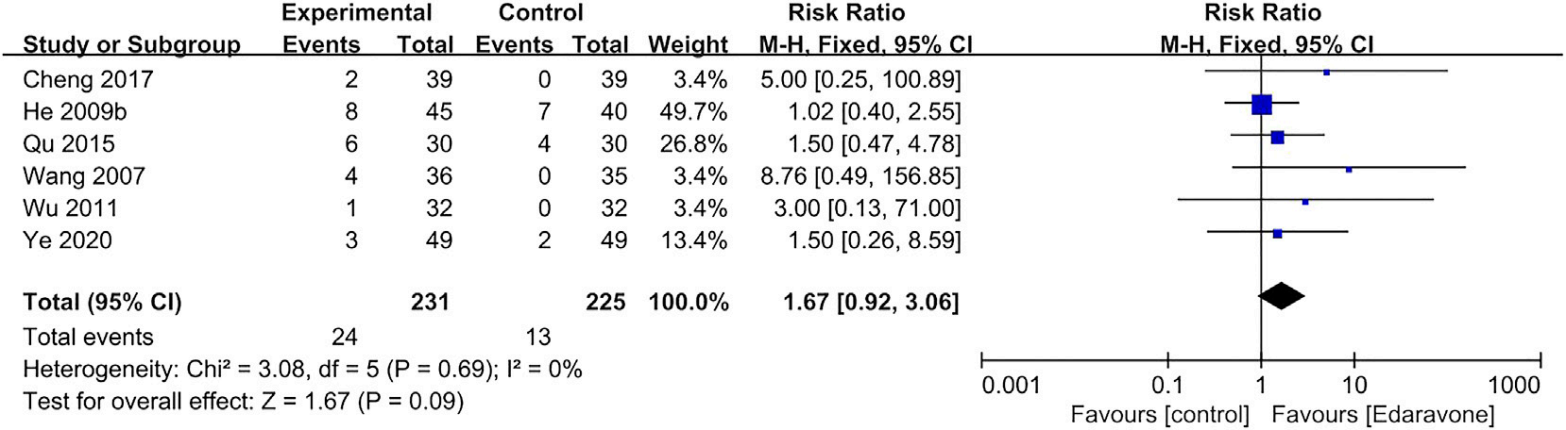
Forest plot for the effect of edaravone on adverse events.

In all studies, the data on adverse events were too disparate and inconsistently reported to allow for further formal analysis. A total of six articles (Wang et al., 2007; He et al., 2009b; Wu, 2011; Qu and Wei, 2015; Cheng, 2017; Ye et al., 2020) reported adverse events, and there was no significant difference between the experimental group and the control group [RR = 1.67; 95% CI (0.92–3.06); *p* = 0.09; *I* ^*2*^ = 0%] (Figure 8). A total of 24 participants developed adverse events during or after edaravone treatment, and the most frequently reported adverse events were kidney impairment, liver impairment, and skin irritation.

### 3.5 Bias of publication

**FIGURE 9:**
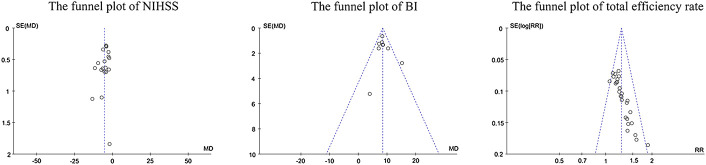
Funnel plots of the NIHSS score, BI, and total efficiency rate.

Visual inspection of funnel plots did not reveal marked asymmetry, suggesting no significant publication bias for the effect of edaravone on the NIHSS score and BI. The distribution of scatter points regarding the total efficiency rate was asymmetric, suggesting publication bias might be present (Figure 9). Egger’s test showed no obvious publication bias risk in the NIHSS score (*p* > 0.05) and BI (*p* > 0.05), while it showed high bias risk in the total efficiency rate (*p* = 0.012).

### 3.6 Risk of bias and grading of recommendations assessment, development and evaluation assessment

No study was rated as having a low risk of bias, while some showed a high risk of bias or near high risk of bias. The certainty of evidence was rated as low for all-cause mortality, activities of daily living, hematoma volume, total efficiency rate, and safety outcomes, while it was rated very low for neurologic deficits, which meant that confidence in the effect size estimates was very limited. The main reasons for downgrading the evidence were risk of bias, inconsistency, and imprecision (Supplementary Figure S9).

## 4 Discussion

This study did not favor edaravone for reducing all-cause mortality compared with placebo or added on co-interventions. Simultaneously, we could not draw a conclusion regarding the effects of edaravone on long-term functional outcomes owing to the lack of corresponding data. We are supposed to attach great importance to the aforementioned two outcomes, as acute ICH is a medical emergency that endangers patients’ lives. Moreover, edaravone is a relatively expensive medicine, costing approximately 600–860 US dollars for one standard course of treatment per stroke patient in China (Yang et al., 2011). Irrational use of edaravone may bring an enormous economic burden to patients and society.

As for secondary outcomes, very low-certainty evidence supported that edaravone could alleviate neurological deficits. Low-certainty evidence revealed that edaravone could improve the activities of daily living and reduce hematoma volume. Heterogeneity was substantial for the NIHSS score and hematoma volume. Although we attempted to perform subgroup analyses by severity, co-intervention, duration of treatment, and dose range of edaravone per day to explore the heterogeneity source, the heterogeneity did not decrease. In addition, low-certainty evidence suggested that no difference existed between the two groups regarding safety outcomes. The reported adverse reactions (including kidney impairment, liver impairment, skin irritation, nausea, vomiting, pruritus, and palpitations) may be related to edaravone or other therapeutic agents or procedures. Compared with previous systematic reviews and meta-analyses, our study attempted to explore whether edaravone could reduce mortality or not (Yang et al., 2011; Yang et al., 2015). Consistent with previous studies revealing that edaravone could improve short-term neurological deficits, we confirmed this finding numerically. However, caution should be paid in interpreting these results. Among the 14 studies included in the systematic review and meta-analysis published in 2015, only 10 studies overlapped with the 38 studies included in this study. Although we attempted to contact the authors, the random methods were still unclear in the four studies; therefore, we did not include them (Pang and Li, 2007; Cen, 2008; Sang et al., 2008; Shi et al., 2008). In addition, the heterogeneities of the original studies were too high to be reduced by sensitivity and subgroup analyses. After reading the full text, we used the random effects model to pool data in view of clinical similarity. We suggest being cautious in understanding and interpreting the results.

Some articles reported the total efficiency rate, which could be improved by edaravone *via* pooling the corresponding data. Compared with other outcomes, the total efficiency rate remains controversial since the essence of surrogate composite outcome has not been rigorously validated for treatment effect assessment. Objective bias was unavoidable when selecting and setting standards. Concurrently, pooling data may increase the probability of error (Armstrong and Westerhout, 2017; McCoy, 2018).

In conclusion, the current systematic review and meta-analysis revealed that no association was found between the edaravone intervention and all-cause mortality in patients with ICH when initiated within 7 days of symptom onset. Although the outcomes showed that edaravone was effective in alleviating neurological impairment, improving the activities of daily living, and reducing hematoma volume, they were summarized with significant statistical heterogeneity and acted as surrogate outcomes in terms of assessment of specific treatment for acute ICH.

Our review has some potential limitations. The included trials varied in several aspects, such as patient characteristics, co-interventions, and follow-up period. Substantial heterogeneity was observed, which lowered the evidence grade. Despite the significant statistical heterogeneity, we still pooled the data of the NIHSS score and hematoma volume because we found acceptable clinical heterogeneity in terms of age, sex, onset time, amount of bleeding, and lesion sites. Furthermore, only one study reported long-term functional outcomes measured with the BI at 60 days. However, we had to exclude this study owing to the undescribed sequence generation and ICH onset time even though we contacted the authors *via* email and telephone. Moreover, nearly half of the included trials were at high risk of bias, which mainly resulted from unreported allocation concealment, blinding of participants and personnel, and blinding of outcome assessment. The faulty methodology brought about selection bias, performance bias, and detection bias. Future studies should overcome these shortcomings. To improve research quality and reduce associated bias, future studies should focus on the implementation of allocation concealment, including central randomization, and sequentially numbered, opaque, sealed envelopes. Furthermore, the research is supposed to report rigorously whether the blinding method is used. If done, it had better provide specific information on the implementation of the blinding method and characteristics of drug consistency. Most studies were conducted based on co-interventions rather than the placebo control. The efficacy of a single therapy may be better assessed using the placebo control. In addition, future research should focus on exploring the association between edaravone and other clinical measures that have been proven to affect patient mortality and quality of life. Given that no study has been performed outside the Chinese mainland, the aforementioned results of limited generalizability should be interpreted cautiously when used for reference to other countries in the future.

## 5 Conclusion

In this review, edaravone was not associated with mortality reduction when initiated within 7 days of ICH onset. The effect of edaravone on long-term functional outcomes remained unknown. Although the pooled data showed that edaravone could alleviate neurological deficits, improve activities of daily living, and reduce hematoma volume, the interpretation of these results still required particular caution. Presently, the results are insufficient to support edaravone as a routine treatment option for acute ICH. The conclusive efficacy and safety of edaravone for the treatment of acute ICH need to be further validated by rigorous studies to update the evidence.

## Data Availability

The original contributions presented in the study are included in the article/Supplementary Material; further inquiries can be directed to the corresponding authors.

## Abbreviations

AP, apurinic/apyrimidinic; BI, Barthel index; CI, confidence interval; CNKI, China National Knowledge Infrastructure; CNS, Canadian Neurological Scale; ESS, European Stroke Scale; GOS, Glasgow Outcome Scale; ICH, intracerebral hemorrhage; MD, mean difference; MESSS, Modified Edinburgh-Scandinavian Stroke Scale; mRS, Modified Rankin Scale; NIHSS, National Institutes of Health Stroke Scale; RCTs, randomized controlled trials; RR, relative risk; SSS, Scandinavian Stroke Scale; and VIP, Chinese VIP information
