# Supplementary material for: Edaravone use in acute intracerebral hemorrhage: A systematic review and meta-analysis of randomized controlled trials
Source: Front Pharmacol. 2022 Aug 12;13:935198. doi: 10.3389/fphar.2022.935198 (PMC9412023; doi:10.3389/fphar.2022.935198)

Supplementary Files

**Table 1** Search Strategy Example: PubMed search

**Figure 1** Forest plot of subgroup analysis by severity for the effect of edaravone on NIHSS score (moderate-severe, severe)

**Figure 2** Forest plot of subgroup analysis by co-intervention for the effect of edaravone on NIHSS score (conventional treatment, conventional treatment plus surgery, other co-interventions)

**Figure 3** Forest plot of subgroup analysis by duration of treatment for the effect of edaravone on NIHSS score (14 days, 28 days, 30 days)

**Figure 4** Forest plot of subgroup analysis by dose range per day for the effect of edaravone on NIHSS score (30mg/d, 60mg/d)

**Figure 5** Forest plot of subgroup analysis by severity for the effect of edaravone on hematoma volume (moderate-severe, severe)

**Figure 6** Forest plot of subgroup analysis by co-intervention for the effect of edaravone on hematoma volume (conventional treatment, conventional treatment plus surgery, other co-interventions)

**Figure 7** Forest plot of subgroup analysis by duration of treatment for the effect of edaravone on hematoma volume (14 days, 30 days)

**Figure 8** Forest plot of subgroup analysis by dose range per day for the effect of edaravone on hematoma volume (30mg/d, 60mg/d)

**Figure 9** Grading of Evidence

**Table 1** Search Strategy Example: PubMed search

| **No** | **Search items** |
| --- | --- |
| 1 | Cerebral Hemorrhage |
| 2 | Hemorrhage, Cerebrum |
| 3 | Cerebrum Hemorrhage |
| 4 | Cerebrum Hemorrhages |
| 5 | Hemorrhages, Cerebrum |
| 6 | Cerebral Parenchymal Hemorrhage |
| 7 | Cerebral Parenchymal Hemorrhages |
| 8 | Hemorrhage, Cerebral Parenchymal |
| 9 | Hemorrhages, Cerebral Parenchymal |
| 10 | Parenchymal Hemorrhage, Cerebral |
| 11 | Parenchymal Hemorrhages, Cerebral |
| 12 | Intracerebral Hemorrhage |
| 13 | Intracerebral Haemorrhage |
| 14 | Hemorrhage, Intracerebral |
| 15 | Hemorrhages, Intracerebral |
| 16 | Intracerebral Hemorrhages |
| 17 | Hemorrhage, Cerebral |
| 18 | Cerebral Hemorrhages |
| 19 | Hemorrhages, Cerebral |
| 20 | Brain Hemorrhage, Cerebral |
| 21 | Brain Hemorrhages, Cerebral |
| 22 | Cerebral Brain Hemorrhage |
| 22 | Cerebral Brain Hemorrhages |
| 23 | Hemorrhage, Cerebral Brai |
| 24 | Hemorrhages, Cerebral Brain |
| **25** | **1 OR 2-24** |
| 26 | Edaravone |
| 27 | Norantipyrine |
| 28 | Norphenazone |
| 29 | Edarabone |
| 30 | 1-Phenyl-3-methyl-5-pyrazolone |
| 31 | 1 Phenyl 3 methyl 5 pyrazolone |
| 32 | 3-Methyl-1-phenyl-2-pyrazolin-5-one |
| 33 | 3 Methyl 1 phenyl 2 pyrazolin 5 one |
| 34 | MCI 186 |
| 35 | MCI-186 |
| 36 | MCI186 |
| 37 | Radicava |
| 38 | Phenylmethylpyrazolone |
| **39** | **26 OR 27-38** |
| 40 | Randomized controlled trial |
| 41 | Controlled clinical trial |
| 42 | Randomized |
| 43 | Placebo |
| 44 | randomly |
| **45** | **40-44** |
| **46** | **25 AND 39 AND 45** |

**Figure 1** Forest plot of subgroup analysis by severity^*^ for the effect of edaravone on NIHSS score (moderate-severe, severe)


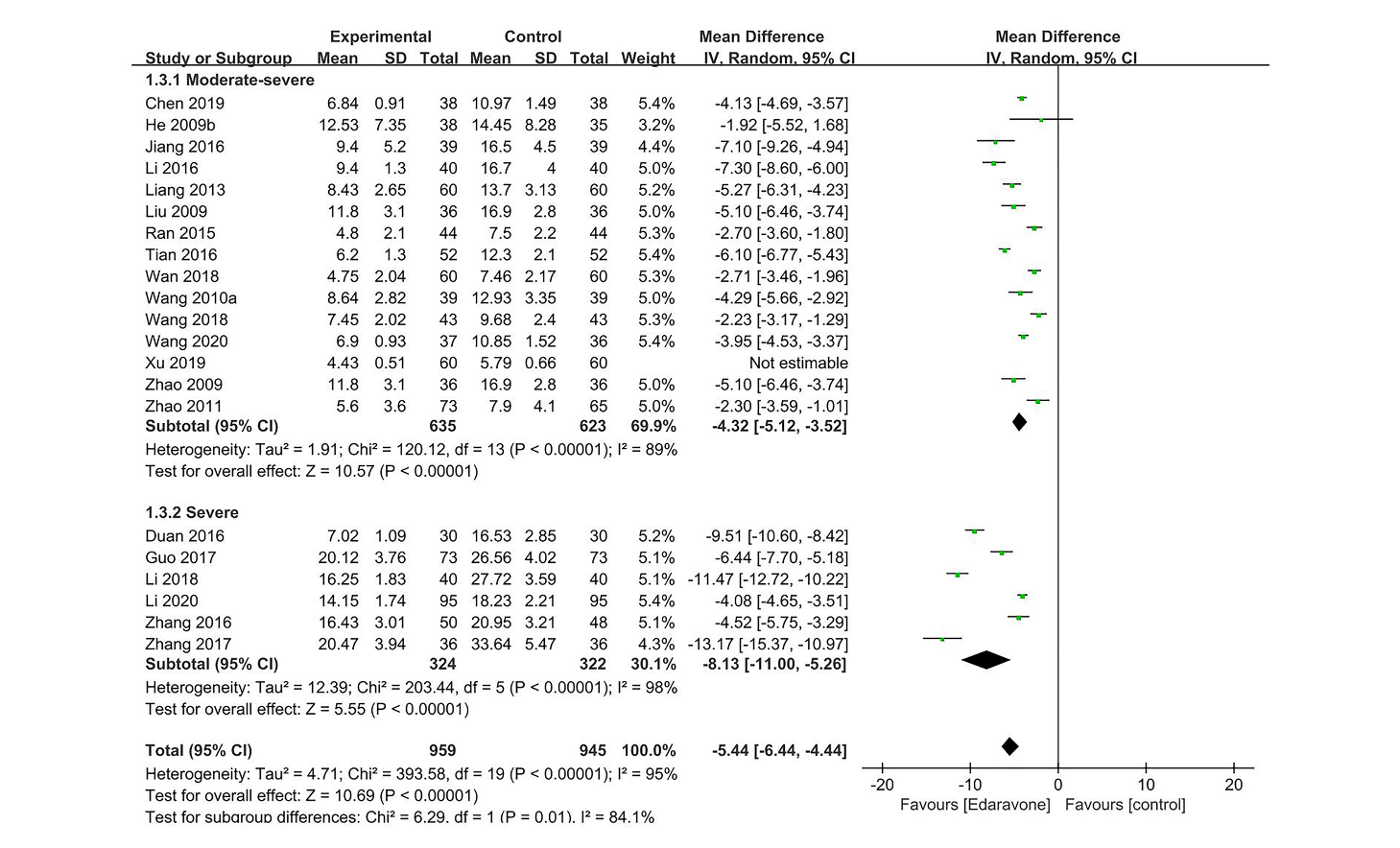


^*^ Severity was assessed by NIHSS score before treatment, and divided into two groups.

moderate-to-severe: NIHSS score 5-25 before treatment

severe: NIHSS score above 25 before treatment

**Figure 2** Forest plot of subgroup analysis by co-intervention for the effect of edaravone on NIHSS score (conventional treatment, conventional treatment plus surgery, other co-interventions)


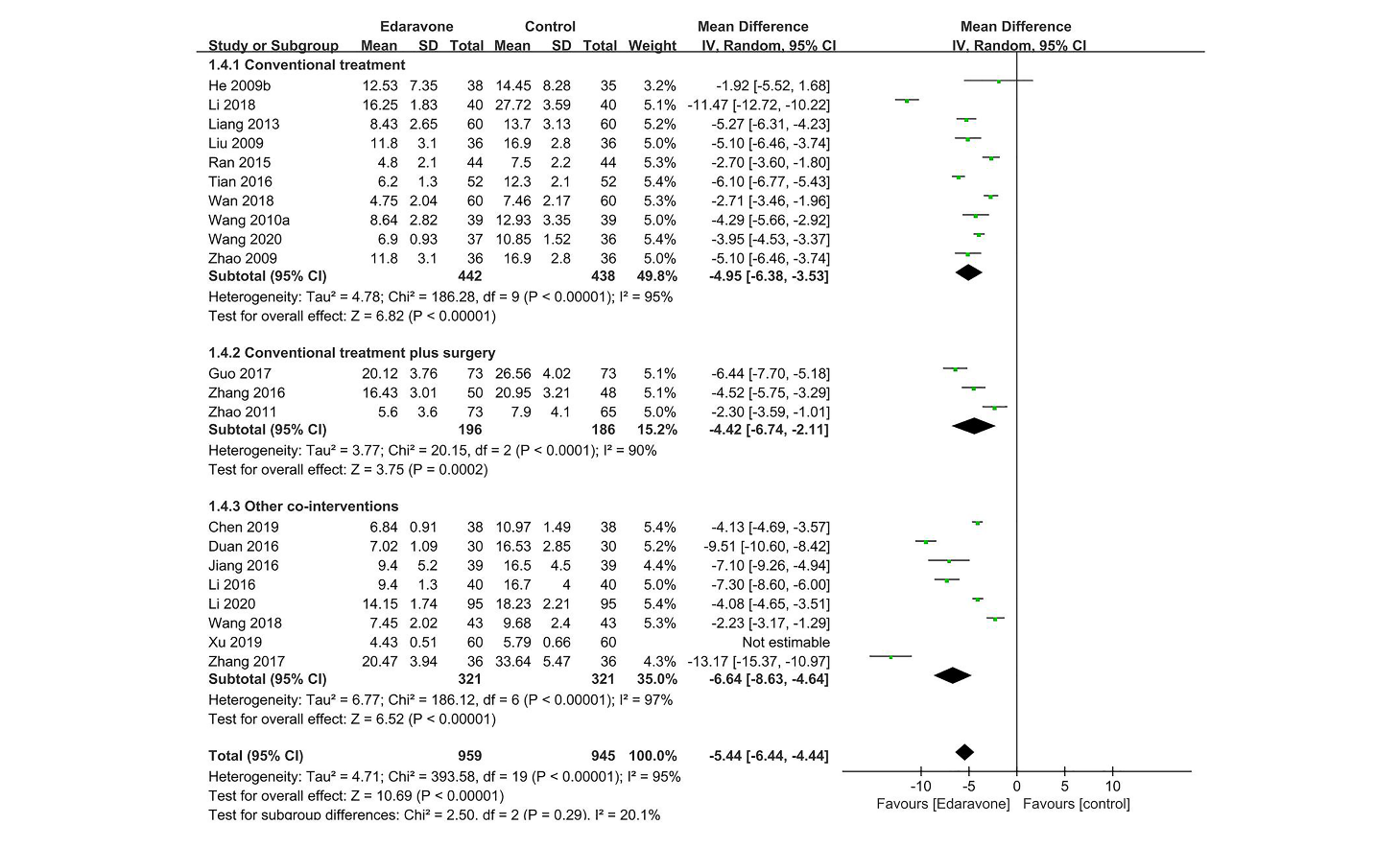


**Figure 3** Forest plot of subgroup analysis by duration of treatment for the effect of edaravone on NIHSS score (14 days, 28 days, 30 days)


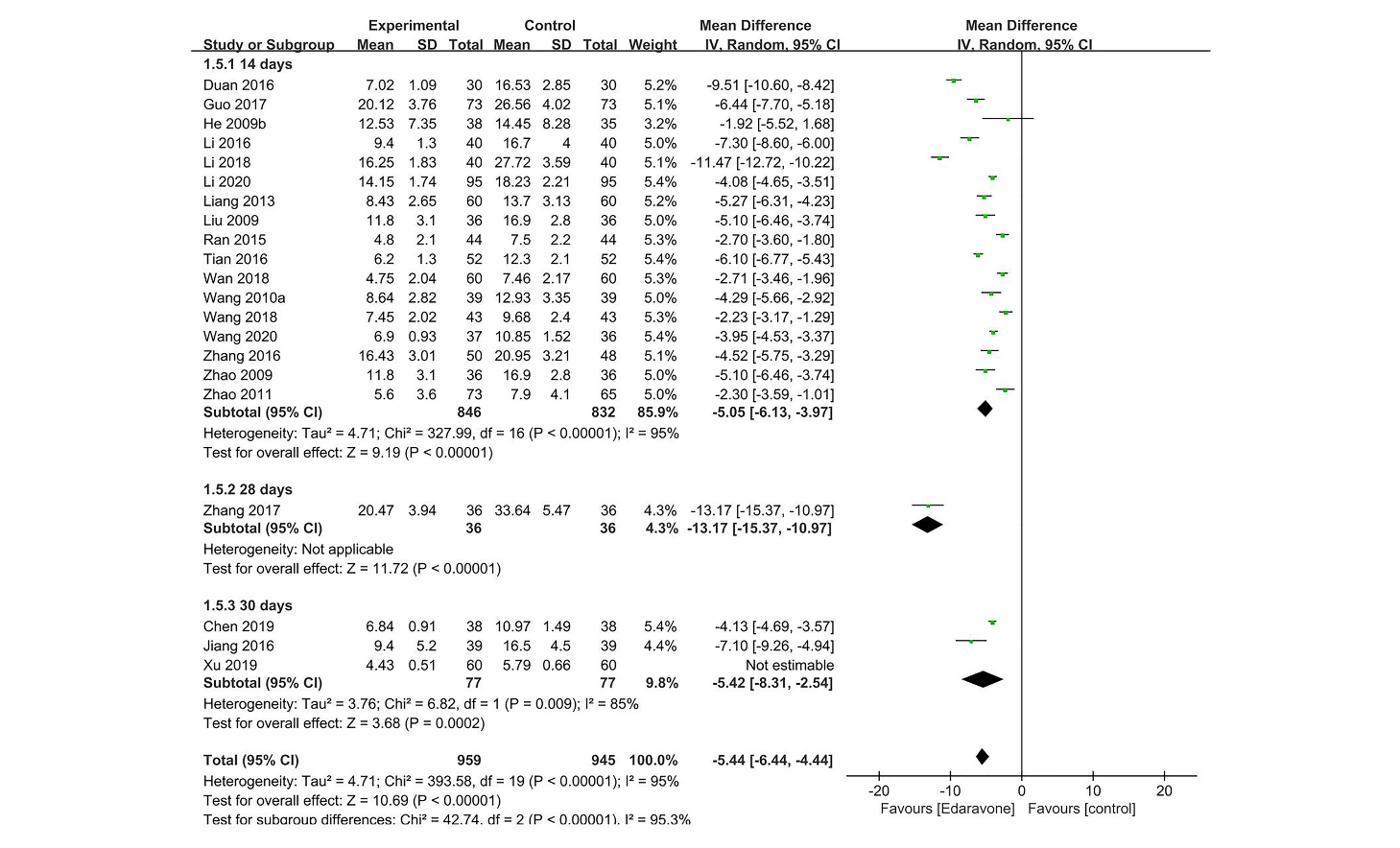


**Figure 4** Forest plot of subgroup analysis by dose range per day for the effect of edaravone on NIHSS score (30mg/d, 60mg/d)


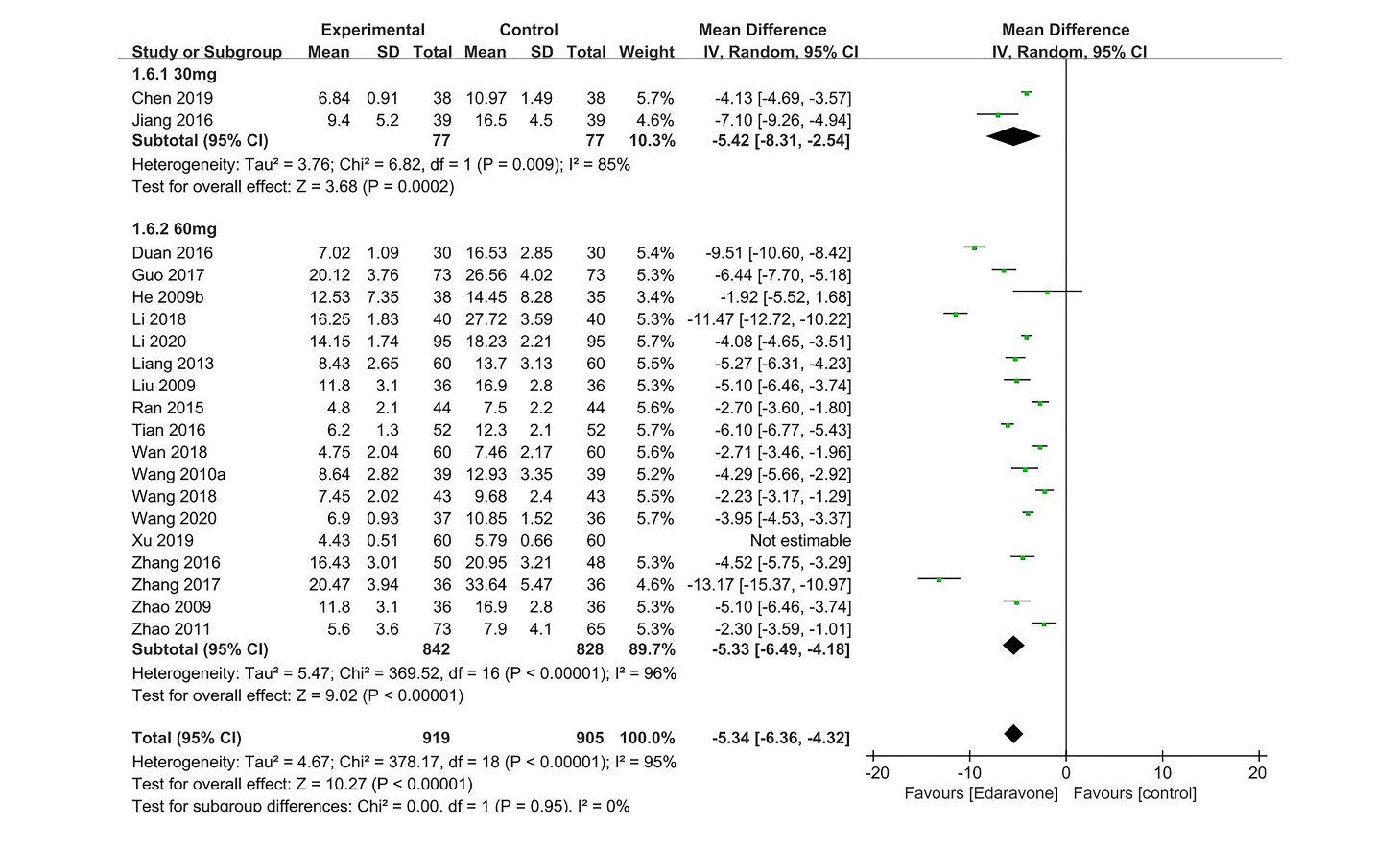


**Figure 5** Forest plot of subgroup analysis by severity^*^ for the effect of edaravone on hematoma volume (moderate-severe, severe)


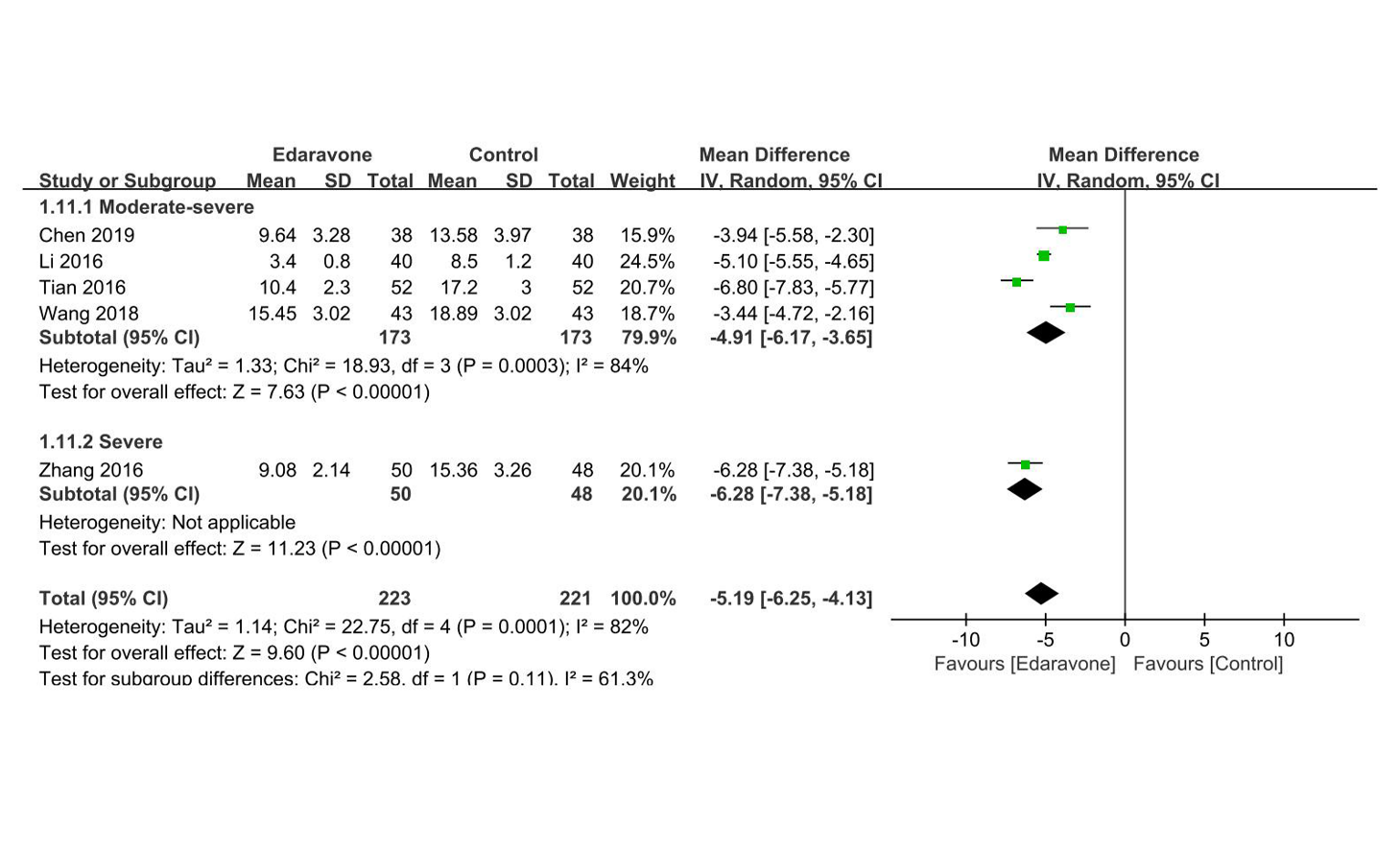


^*^ Severity was assessed by NIHSS score before treatment, and divided into two groups.

moderate-to-severe: NIHSS score 5-25 before treatment

severe: NIHSS score above 25 before treatment

**Figure 6** Forest plot of subgroup analysis by co-intervention for the effect of edaravone on hematoma volume (conventional treatment, conventional treatment plus surgery, other co-interventions)


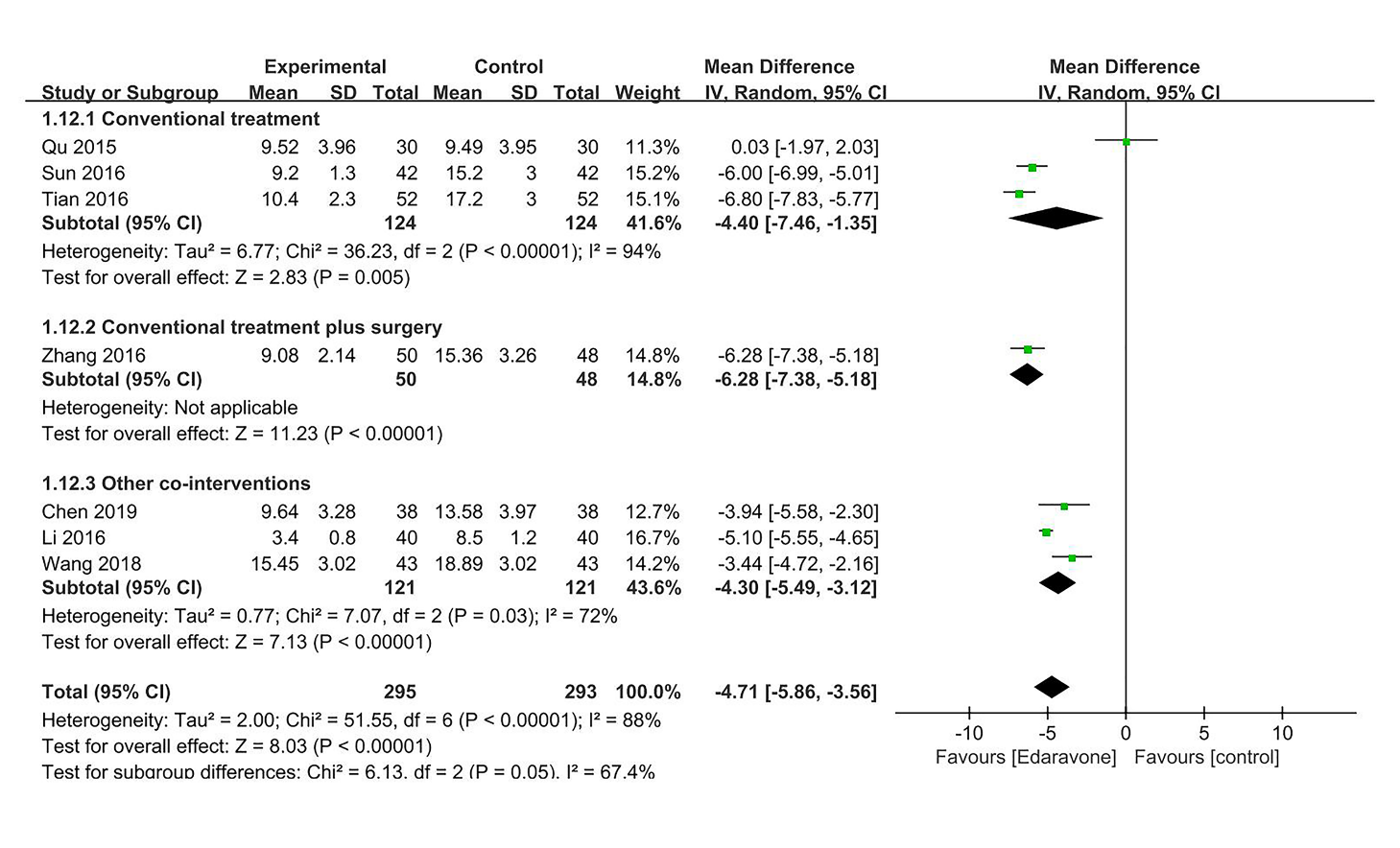


**Figure 7** Forest plot of subgroup analysis by duration of treatment for the effect of edaravone on hematoma volume (14 days, 30 days)


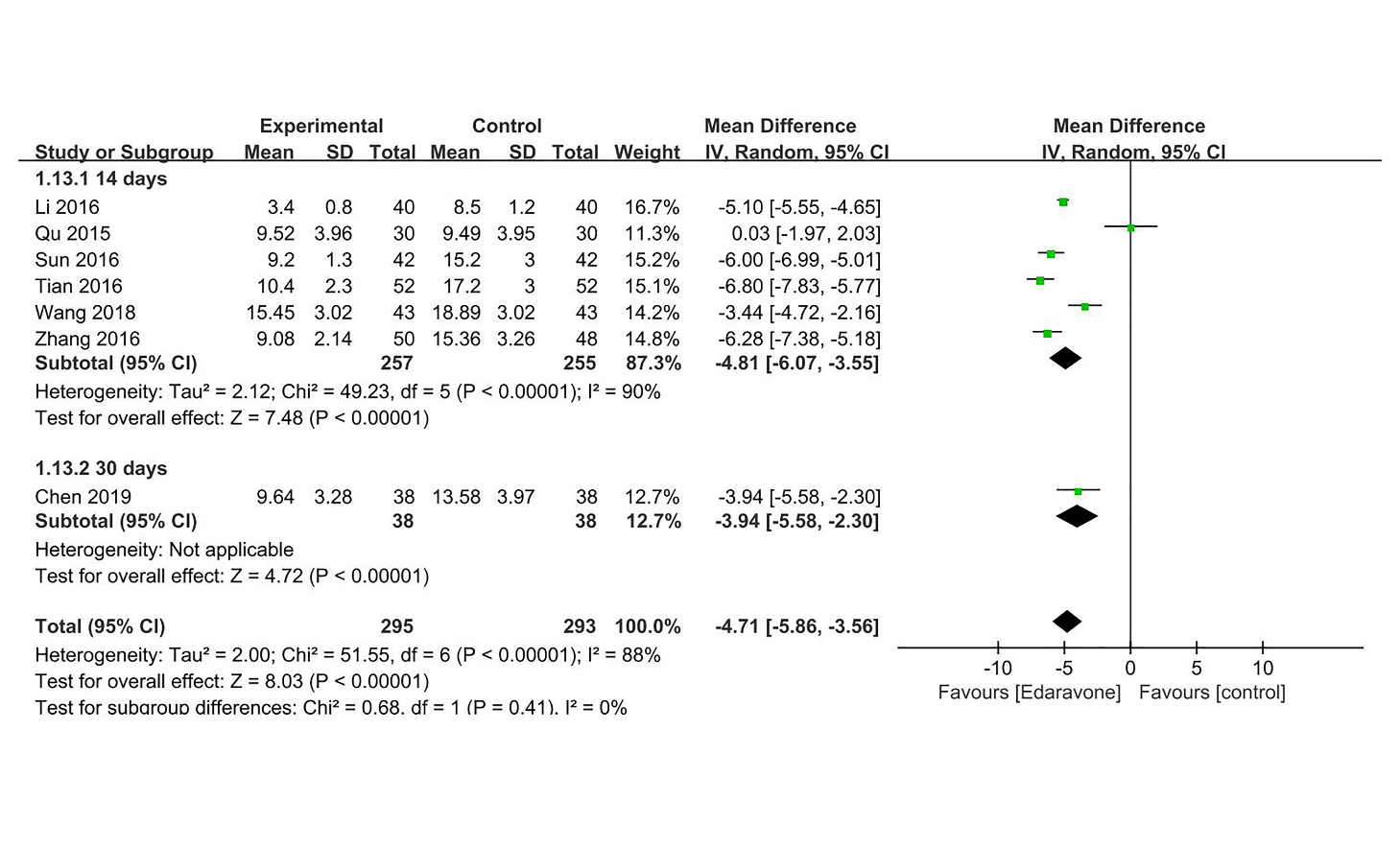


**Figure 8** Forest plot of subgroup analysis by dose range per day for the effect of edaravone on hematoma volume (30mg/d, 60mg/d)


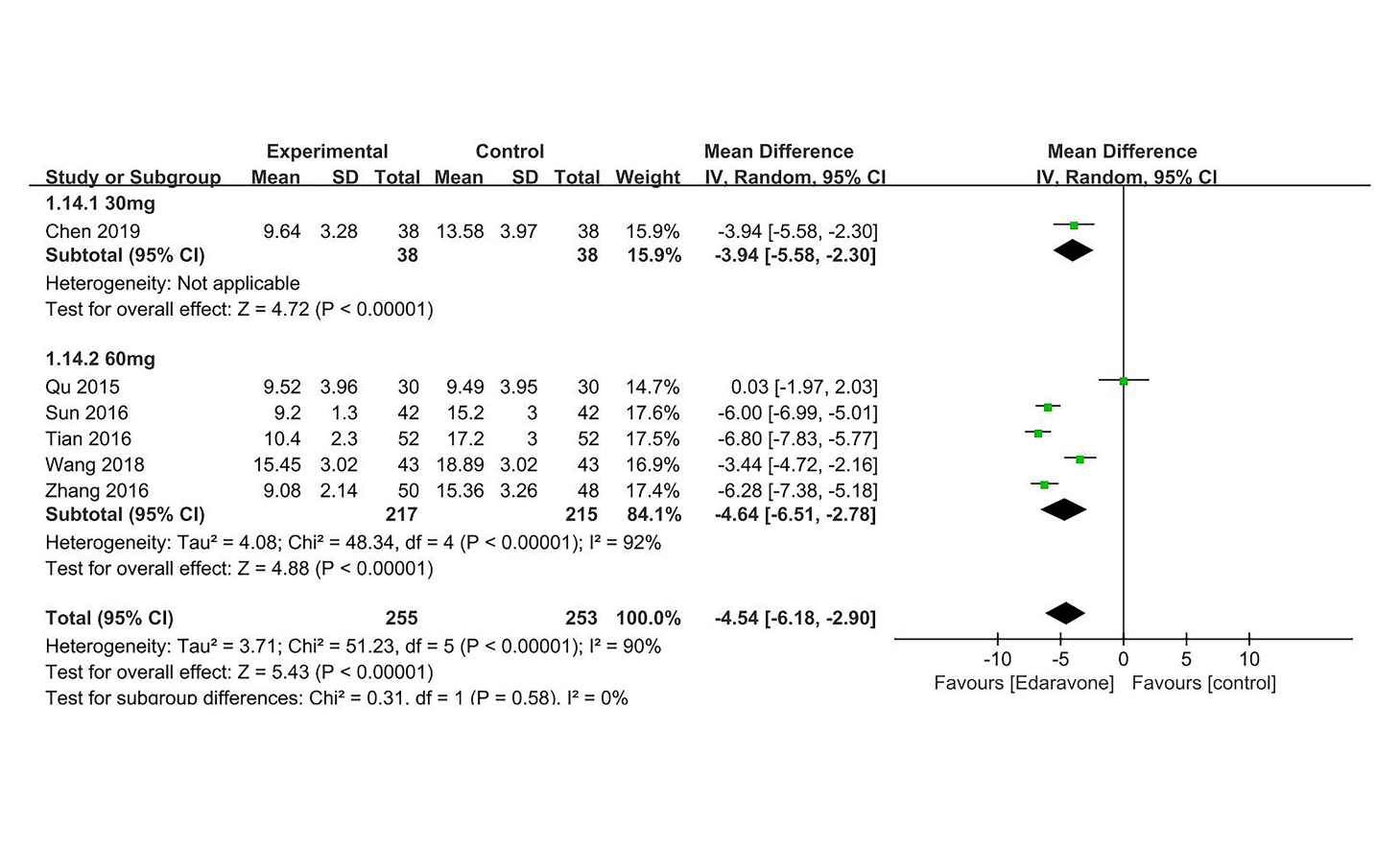


**Figure 9** Grading of Evidence


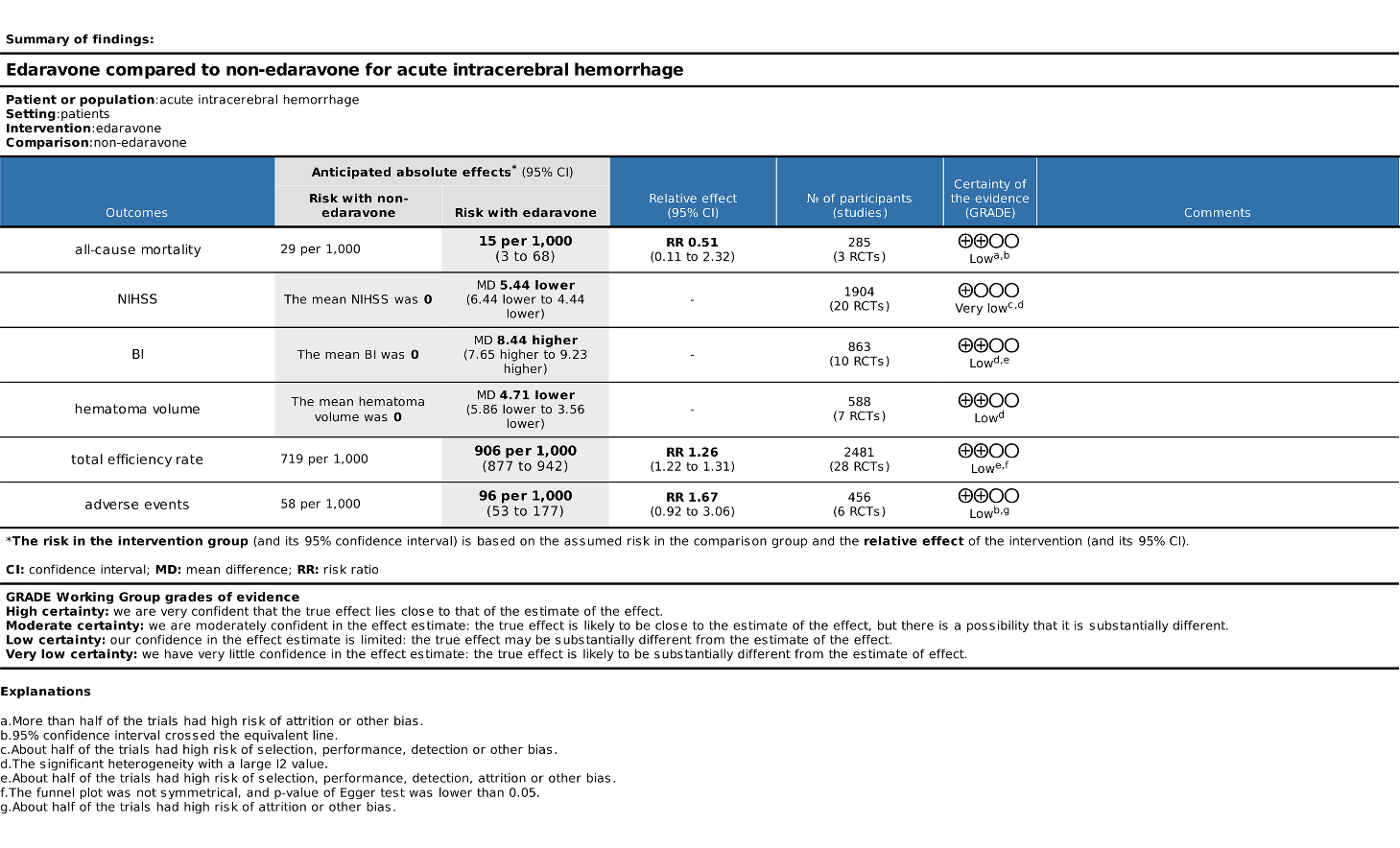

Supplement: Supplementary file 1 [file DataSheet1.docx]
